# Widely Targeted Metabolomics Analysis Reveals Key Quality-Related Metabolites in Kernels of Sweet Corn

**DOI:** 10.1155/2021/2654546

**Published:** 2021-02-08

**Authors:** Ruichun Yang, Yunfeng Li, Yuanyuan Zhang, Jun Huang, Junjie Liu, Zimei Lin, Qinqin Yu, Aimin Wu, Bo Wang

**Affiliations:** ^1^Guangdong Key Laboratory of Plant Molecular Breeding, College of Agriculture, South China Agricultural University, 510642 Guangdong, China; ^2^State Key Laboratory for Conservation and Utilization of Subtropical Agro-Bioresources, South China Agricultural University, 510642 Guangzhou, China; ^3^Guangdong Key Laboratory for Innovative Development and Utilization of Forest Plant Germplasm, College of Forestry and Landscape Architectures, South China Agricultural University, 510642 Guangdong, China

## Abstract

Sweet corn (*Zea mays convar. saccharata* var. *rugosa*) is a major economic vegetable crop. Different sweet corn cultivars vary largely in flavor, texture, and nutrition. The present study performed widely targeted metabolomics analysis based on the HPLC-MS/MS technology to analyze the metabolic profiles in three sweet corn cultivars widely grown in China. A total of 568 metabolites in the three sweet corn cultivars were detected, of which 262 differential metabolites significantly changed among cultivars. Carbohydrates, organic acids, and amino acids were the majority detected primary metabolites. Organic acids were mainly concentrated on shikimate, benzoic acids, and quinic acid with aromatic groups. And the essential amino acids for the human body, methionine and threonine, were highly accumulated in the high-quality cultivar. In addition, phenylpropanoids and alkaloids were the most enriched secondary metabolites while terpenes were low-detected in sweet corn kernels. We found that the flavonoids exist in both free form and glycosylated form in sweet corn kernels. PCA and HCA revealed clear separations among the three sweet corn cultivars, suggesting distinctive metabolome profiles among three cultivars. The differential metabolites were mapped into flavonoid biosynthesis, phenylpropanoid biosynthesis, biosynthesis of amino acids, and other pathways according to the KEGG classification. Furthermore, we identified skimmin, N′,N^″^-diferuloylspermidine, and 3-hydroxyanthranilic acid as the key quality-related metabolites related to grain quality traits in sweet corn. The results suggested variations of metabolic composition among the three cultivars, providing the reference quality-related metabolites for sweet corn breeding.

## 1. Introduction

Corn (*Zea mays*) is a member of the *Poaceae* family, with field corn and sweet corn as two main types. Matured field corn is used as livestock feed, ethanol, cereal, and processed food products, while immature sweet corn is harvested and used as vegetable and fruit [1]. Sweet corn is derived from the endosperm mutant of the cultivated corn [2, 3]. Its kernels have high sugar content at the milk stage, making them widely grown in the context of increasing popularity worldwide. Various cultivars of sweet corn have different grain quality, mainly due to their evaluation criteria during the breeding process. These standard quality-related criteria are sweet, tender pericarp, and long-lasting quality, which ignores the key quality-related nutrient components.

Metabolomic profiling is a key strategy to analyze the nutrient components at a molecular level. Currently, the metabolomic platform is composed of separation technologies and detection technologies. The separation technologies include liquid chromatography (LC) and gas chromatography (GC). LC is generally used to separate global molecules, while GC is only suitable for volatiles or some nonvolatile substances following derivatization [4]. The detection technologies include mass spectrometry (MS) and nuclear magnetic resonance (NMR) spectroscopy [5, 6]. Compared to NMR, MS offers high sensitivity and balanced selectivity for the detection of picomole to femtomole levels of many primary and secondary metabolites [4]. Thus, global metabolite profiling analysis based on HPLC-MS/MS could detect as many metabolites as possible in sweet corn.

The cultivated field corns show biochemical diversity in kernels. More than 150 metabolites have been quantified in maize kernels of the recombinant inbred by metabolome-based genome-wide association study and metabolic quantitative trait locus study, respectively [7–9], indicating the metabolic diversity of maize kernels. Flavonoids, polyamine, phenylpropanoids, and free amino acids are the major components in common field maize kernels [7–10]. In addition, water-soluble sugars, organic acids, and amino acids were characterized in sweet corn kernels by GC-MS and NMR [11]. Compared to field corn, sweet corn lines showed dramatic metabolic differences in seed composition, most likely due to the abnormal starch content [11]. However, few studies have reported metabolome analysis of secondary metabolites for sweet corn kernels.

As important fresh-edible vegetables, the nutrients and bioactive compounds in sweet corn are also the major attraction in addition to the texture and taste. Thus, a widely targeted metabolic analysis was conducted in the present study to investigate metabolic variations and quality-related metabolites in three corn cultivars by using HPLC-MS/MS. In addition, we identified skimmin, N′,N^″^-diferuloylspermidine, and 3-hydroxyanthranilic acid as the key quality-related metabolites for high-quality sweet corn breeding. To the best of our knowledge, this is the first study that used the HPLC-MS-based metabolomic approach to reveal the insight into the metabolic variation in sweet corn kernels.

## 2. Materials and Methods

### 2.1. Plant Materials

In this study, we used three representative sweet corn cultivars, which were widely cultivated in China and showed high edible quality with high sugar content or thin pericarp: Cupola (CPL), Jinbaitian (JBT), and Jinzhongyu (JZY). These cultivars were cultivated in the experimental fields in South China Agricultural University, Guangzhou (23°17′33^″^N; 113°51′3^″^E), in 2018. The pollination was carried out in May with temperatures of 35 and 25°C (day/night) and relative humidity of 70% for one week. Each cultivar was, respectively, self-pollinated with parchment pollination bags. Kernels of the middle ear from the different positions of the experimental fields were collected at 22 days after pollination. In total, 30 biological replications were collected for each cultivar, every ten of which were finally pooled to give one pooled sample before the extraction of metabolites. The pooled samples were immediately frozen in liquid nitrogen and stored at -80°C for subsequent experiments.

### 2.2. Measurement of Sucrose, Glucose, and Fructose

The ground samples (100 mg) were dissolved in 1 mL ultrapure water at 95°C for 10 min, and the supernatant was collected by centrifugation. The contents of sucrose, glucose, and fructose were detected using ultraviolet spectrophotometry (MAPADA, UV-1200), respectively. For the fructose determination, we added resorcinol to form a characteristic absorption peak at 480 nm. For the sucrose determination, the reductive carbohydrate was destroyed by coheating the samples with alkali. Then, the sucrose was dehydrated into glucose and fructose in acidic conditions. The absorbance was detected at 480 nm; due to that, the dehydrated fructose reacted with resorcinol. For glucose determination, we oxidized the glucose into gluconic acid and hydrogen peroxide. The peroxidase was added to catalyze hydrogen peroxide and 4-aminoantipyrine coupled phenol at 25°C, forming a characteristic absorption peak at 505 nm. The soluble sugar contents were calculated according to the fresh weight of the sample.

### 2.3. Measurement of Pericarp Thickness

Scanning electron microscopy (SEM, Zeiss EVO MA 15) was used to observe the pericarp thickness. Fresh sweet corn kernels were frozen in liquid nitrogen and cut from the top of the seeds. They were fixed with 2.5% glutaraldehyde at 4°C and washed twice with 0.2 mol/L phosphate buffer saline (PBS) for 10 min each time. Afterward, the samples were fixed with 1% citric acid at 4°C for 2 h and washed three times with PBS for 10 min each time. A volume fraction of 30%, 50%, 70%, 90%, and 100% ethanol solution was used for dehydration. After drying, the samples were coated and photographed by a scanning electron microscope at an accelerating voltage of 15 kV. Each experiment was replicated three times with different kernels.

### 2.4. Measurement of C4H, CAD, and CHI Activities

The enzyme activity assay kits (Suzhou Comin Biotechnology Co., Ltd., China) were used to detect cinnamic acid-4-hydroxylase (C4H), cinnamyl-alcohol dehydrogenase (CAD), and chalcone isomerase (CHI) in sweet corn kernels following the manufacturer's instructions. C4H catalyzes cinnamic acid and NADP into 4-coumarate and NADPH, and CAD catalyzes the formation of cinnamaldehyde and NADPH from cinnamyl alcohol and NADP; thus, the rate of NADPH generation at 340 nm was detected to reflect the C4H and CAD activity, respectively. In addition, CHI catalyzes the cyclization of chalcone to form 4,5,7-trihydroxyflavanone, which showed the absorbance at 381 nm. Then, the absorbances of each enzyme were detected using the ultraviolet spectrophotometer before 30 minutes of preheating. Finally, the enzyme activity values of each enzyme were calculated. Three replicates of each sample were performed.

### 2.5. Sample Preparation and Metabolite Extraction

Freeze-dried kernels were ground using a grinder (MM 400, Retsch) at 30 Hz for 1.5 min. The powder (100 mg) was weighed and extracted overnight at 4°C with 1.0 mL 70% aqueous methanol. After centrifugation at 10,000g for 10 min, the extracts were absorbed (CNWBOND Carbon-GCB SPE Cartridge, ANPEL) and filtered by 0.22 *μ*m filters (SCAA-104, ANPEL) for LC-MS analysis.

### 2.6. Metabolic Profiling Analysis

The extracts were analyzed using an HPLC chromatographic system (Shim-pack UFLC SHIMADZU CBM30A) coupled with a triple quadrupole-linear ion trap mass spectrometer (Applied Biosystems 6500 Q TRAP). The HPLC system was equipped with an ACQUITY UHPLC HSS T3 C18 column (Waters, 1.8 *μ*m × 2.1 mm × 100 mm). The chromatographic separation was achieved by using water and acetonitrile (with 0.04% acetic acid for each) as the mobile phase. The elution gradient referred to Chen's method [12, 13]; gradient program was 95 : 5 *v*/*v* at 0 min, 5 : 95 *v*/*v* at 11.0 min, 5 : 95 *v*/*v* at 12.0 min, 95 : 5 *v*/*v* at 12.1 min, and 95 : 5 *v*/*v* at 15.0 min with the 0.40 mL/min of flow rate (Table S2). The column temperature was 40°C, and the injection volume was 2 *μ*L. The effluent was connected to an ESI-triple quadrupole linear ion trap mass spectrometer (ESI-QTRAP-MS).

Linear ion trap (LIT) and triple quadrupole (QQQ) scans were acquired on QTRAP equipped with an ESI Turbo Ion-Spray interface in the positive ion mode and controlled by Analyst 1.6.3 software (AB Sciex). The ESI source operation followed Chen's parameters [12] as follows: ion source, turbo spray; source temperature 550°C; ion spray voltage (IS) 5500 V; ion source gas I (GSI), gas II (GSII), and curtain gas (CUR) were set at 55, 60, and 25.0 psi, respectively; the collision gas (CAD) was high. Instrument tuning and mass calibration were performed with 10 and 100 *μ*mol/L polypropylene glycol solutions in QQQ and LIT modes, respectively. QQQ scans were acquired as multiple reaction monitoring (MRM) experiments with collision gas (nitrogen) set to 5 psi. Declustering potential (DP) and collision energy (CE) for individual MRM transitions were determined with further DP and CE optimization [12]. A specific set of MRM transitions were monitored for each period according to the metabolites eluted within this period.

### 2.7. Qualitative and Quantitative Analysis of Metabolites

Metabolite qualitative and quantitative analyses were carried out via the MRM mode of the QQQ mass spectrometer. In the MRM mode, the first quadrupole searched for precursor ions (parent ions) of target substances. The second quadrupole is the collision chamber with applied voltage and gas. The selected precursor ions in the first quadrupole were fragmented into smaller molecular weight ions via induced ionization in the second quadrupole to form product ions. The optimized DP and CE in the second quadrupole were recorded for each precursor–product ion transition. These product ions enter the third quadrupole to select single-fragment ions with the specific *m*/*z*. These steps lead to obtain the metabolite mass spectrometry and increase precision and repeatability of the qualitative and quantitative analysis.

The qualitative analysis was conducted using the stepwise multiple ion monitoring-enhanced product ions (MIM-EPI) strategy, and the MS2T data were analyzed by comparing the accurate precursor spectral ion (Q1) and product spectral ion (Q3) values, retention time (RT) of mass spectrometry, and fragmentation pattern with the self-built MWDB database (Metware Biotechnology Co., Ltd., Wuhan, China). This MWDB database was built with the availably commercial standard compounds, and the metabolite structure analysis was obtained by referencing existing mass spectrometry databases, including the public database of metabolite information, including MassBank (http://www.massbank.jp), KNAPSAcK (http://www.knapsackfamily.com/KNApSAcK/), Human Metabolome Database (HMDB; http://www.hmdb.ca), MoTo DB (http://www.ab.wur.nl/moto), and METLIN (http://metlin.scripps.edu/index.php) [12]. All metabolites in the commercial database MWDB (Metware Biotechnology Co., Ltd., Wuhan, China) have been classified into different categories, including carbohydrates, organic acids, amino acids, nucleotides and derivatives, phenylpropanoids, alkaloids, flavonoids, and proanthocyanins [12]. The possible redundancy caused by different isotopes, in-source fragmentation, K+, Na+, and NH4+ adduct, and dimerization was removed manually for signal/noise (s/n) > 10 and in-house software written in Perl [12].

For the quantitative analysis, all the obtained mass spectrum peaks were used for area integration. The mass spectral peaks for each metabolite were corrected on the basis of information on metabolite Rt and peak type in order to accurately compare the differences in the content of each metabolite from the different samples.

### 2.8. Statistical Analysis

The metabolic data was initially processed with MassHunter software. Postacquisition was log2-transformed and normalized for statistical analysis to improve normality. The unsupervised hierarchical cluster analysis (HCA) was performed using the agglomeration method of “complete linkage” based on the Euclidean distances of the detected metabolites between cultivars. The processed datasets were imported into SPSS for Windows (Version 18.0, Chicago: SPSS Inc.) for principal component analysis (PCA) and orthogonal partial least squares discriminant analysis (OPLS-DA) to visualize the differentiation between and within different cultivars. The OPLS-DA evaluation method separates the *X* matrix into two matrices: one is linearly related to *Y*, and the other one is orthogonal to *Y*. The *Y*-correlated variable is the prediction component, and the latter variable is the orthogonal component. The parameters of the OPLS-DA evaluation model are *R*^2^*X*, *R*^2^*Y*, and *Q*^2^. The *R*^2^*X* and *R*^2^*Y* values were used to explain the interpretation rate of *X* and *Y* by the model, respectively. The *Q*^2^ value was used to indicate the prediction ability of the model. *Q*^2^ value greater than 0.9 indicates an excellent model, while a value greater than 0.5 indicates an effective model. To validate the supervised model, the best-fitted OPLS-DA model was validated using the permutation test with 200 iterations. The fitting validity and predictive ability of the selected OPLS-DA model were assessed by the parameters *R*^2^*Y* and *Q*^2^, respectively. Variable Importance in Projection (VIP) analysis in the OPLS-DA model was used to identify those metabolites having the highest discrimination potential (VIP score ≥ 1). Differences in the metabolites of sweet corn kernels among different cultivars were determined using Welch's *t*-test (*P* < 0.01) as well.

## 3. Results

### 3.1. Sugar Content and Pericarp Thickness in the Three Sweet Corn Cultivars

**Figure 1:**
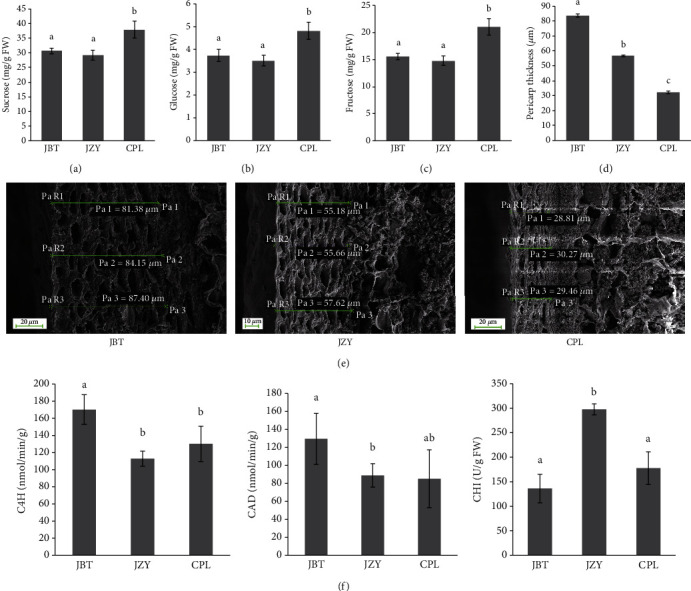
The sugar content and pericarp thickness in three sweet corn cultivars: (a) sucrose content; (b) glucose content; (c) fructose content; (d) pericarp thickness; (e) images of kernel pericarp in sweet corn using a scanning electron microscope. The results were shown as mean ± standard deviation with triplicate experiments for each sample (*P* < 0.05); (f) enzyme activity of C4H, CAD, and CHI in three sweet corn cultivars.

The sugar content and pericarp thickness are two crucial quality factors for sweet corn breeding. We tested these two criteria in the three widely cultivated sweet corns (CPL, JBT, and JZY) in order to confirm the quality difference among these representative sweet corn cultivars in China. The immature fresh kernels obtained at 22 days after pollination were used for determination of sugar content and pericarp thickness. The primary water-soluble saccharides were sucrose, glucose, and fructose in sweet corn [14, 15]. Thus, sucrose, glucose, and fructose contents were determined as the water-soluble saccharide content. CPL was found to have the highest water-soluble saccharide content in the three cultivars, while JBT and JZY shared similar levels of water-soluble saccharides. (Figures 1(a)–1(c)).

The pericarp thickness in the three sweet corn cultivars was observed using SEM. The three sweet corn cultivars showed the differences in thickness. The average pericarp thickness reached to 83.74 *μ*m in JBT, and it was 56.74 *μ*m in JZY. The thin pericarp was detected in CPL, and it was only 32.13 *μ*m (Figures 1(d) and 1(e)). Therefore, we identified CPL as a high-quality sweet corn cultivar. In addition, although there was no significant difference between the water-soluble saccharide content of JZY and JBT, the pericarp thickness was lower in JZY than in JBT (Figures 1(d) and 1(e)). Thus, JZY was identified as medium-quality sweet corn and JBT as a low-quality sweet corn cultivar.

Furthermore, pericarp thickness is contributed by the phenylpropanoids, such as the lignin content. We detected the activities for three enzymes, C4H, CAD, and CHI, in the phenylpropanoid pathway and its branching flavonoid pathway. Results showed that both C4H and CAD activities showed the highest in JBT kernels, which is consistent with its high pericarp thickness (Figures 1(d)–1(f)). The activities of C4H and CAD were low in CPL, and it matches with the reduced pericarp thickness in CPL kernels. The CHI activity was low in CPL as well, indicating the putative variations of phenylpropanoid and flavonoid accumulation in CPL in contrast with JZY and JBT.

### 3.2. The Metabolic Profiling among the Three Sweet Corn Kernels by HPLC-MS/MS

To study the metabolic profiling of kernels in the three sweet corn cultivars (CPL, JBT, and JZY), we performed the metabolomics analysis using HPLC-MS/MS. We conducted PCA and HCA based on the whole dataset to reveal metabolic profiles systematically. The first and second principal components explained 30.54% and 20.11% of the total variation, respectively (Figure S1). PCA suggested a clear separation of these three cultivars. Therefore, the three sweet corn kernels exhibited distinct metabolic profiles among cultivars. In addition, results of HCA (Figure S2) showed all replicates present repeatability well within each cultivar, which is consistent with the PCA result. The metabolome of JBT and JZY was relatively close to each other compared to that of CPL, suggesting metabolic profiles of CPL were prominently different from those of the other two sweet corn cultivars.

### 3.3. Screening and Classification of the Detected Metabolites

**Figure 2:**
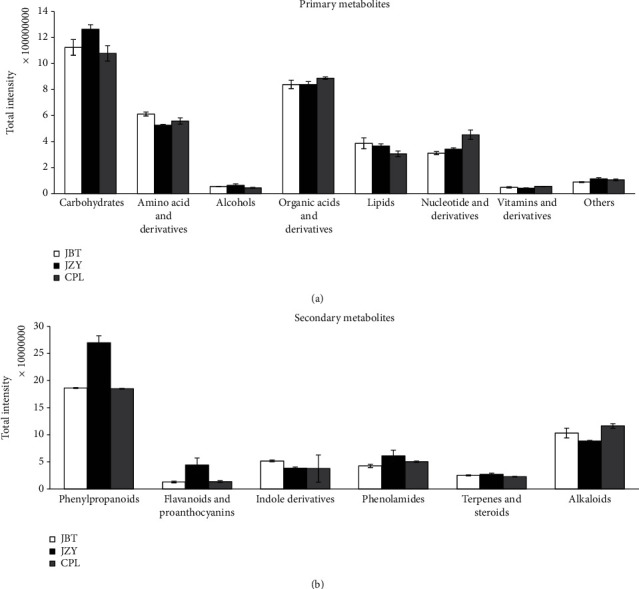
Detected metabolite distribution in sweet corn cultivars: (a) primary metabolites; (b) secondary metabolites.

In total, 568 metabolites were detected by HPLC-MS/MS, representing the broad metabolites in sweet corn. These metabolites were assigned to 14 biochemical categories, including carbohydrates, organic acids, amino acids, nucleotides and derivatives, phenylpropanoids, alkaloids, and flavonoids and proanthocyanins according to the metabolite classification in the MWDB database [12]. These metabolites were grouped into primary metabolites and secondary metabolites (Figure 2). The total intensities of the detected metabolites for some biochemical categories were calculated in each sweet corn cultivars. The composition analysis suggested that carbohydrates, organic acids, and amino acids were the majority of detected primary metabolites in sweet corn kernels (Figure 2(a)). The carbohydrate intensities detected with the highest frequency were maximum in JZY. The intensities of amino acids in JBT were abundant, while the content of detected organic acids, nucleotides, and their derivatives were high in CPL. In addition, phenylpropanoids and alkaloids were the enriched secondary metabolites in sweet corn kernels (Figure 2(b)). Both intensities of phenylpropanoids and flavonoids were high in JZY, and the alkaloids' contents were high in CPL.

### 3.4. Differential Metabolite Analysis in the Sweet Corn's Kernels with Different Quality

Orthogonal partial least-squares discriminant analysis (OPLS-DA) is a better method to discriminate ion peaks that contribute to the classification of samples and to remove noncorrelated variations contained within spectra [16]. OPLS-DA was performed to explore the differential metabolites in pairwise to evaluate the differences between JZY and CPL, JBT and CPL, and JBT and JZY. High *Q*^2^ and goodness of fit (*R*^2^*X*, *R*^2^*Y*) were observed for the pairwise OPLS-DA models between JZY and CPL (*Q*^2^ = 0.921, *R*^2^*X* = 0.506, *R*^2^*Y* = 0.994), JBT and CPL (*Q*^2^ = 0.886, *R*^2^*X* = 0.455, *R*^2^*Y* = 0.997), and JBT and JZY (*Q*^2^ = 0.831, *R*^2^*X* = 0.405, *R*^2^*Y* = 0.995). As shown in Figure S3A, in all the OPLS-DA models, the values of *Q*^2^ were no less than 0.5, and the difference between *R*^2^*Y* and *Q*^2^ was no more than 0.2, indicating excellent quality of our models. The predictive (p1) and orthogonal (o1) component were used (*R*^2^*X* for p1 = 0.506, *R*^2^*X* for o1 = 0.152) for OPLS-DA score plots. Predictive component p1 indicated 50.6% variation in *X*, resulting in separation of the samples into two distinct groups between JZY and CPL (Figure S3B). The score plots showed relatively good separation of samples between JBT and JZY (45.5%) and between JBT and CPL (40.5%) as well. OPLS-DA S-plots were constructed further. The variables present furthest from the center on the arms in the S-plot, indicating the distinctive compounds for each group (Figure S3C). A permutation test with 200 times repeats was performed to verify the OPLS-DA model. *Q*^2^ and *R*^2^*Y* values from the permutation test for the OPLS-DA model were confirmed to be higher than their original values, indicating the validity and suitability of this model (Figure S3D).

**Figure 3:**
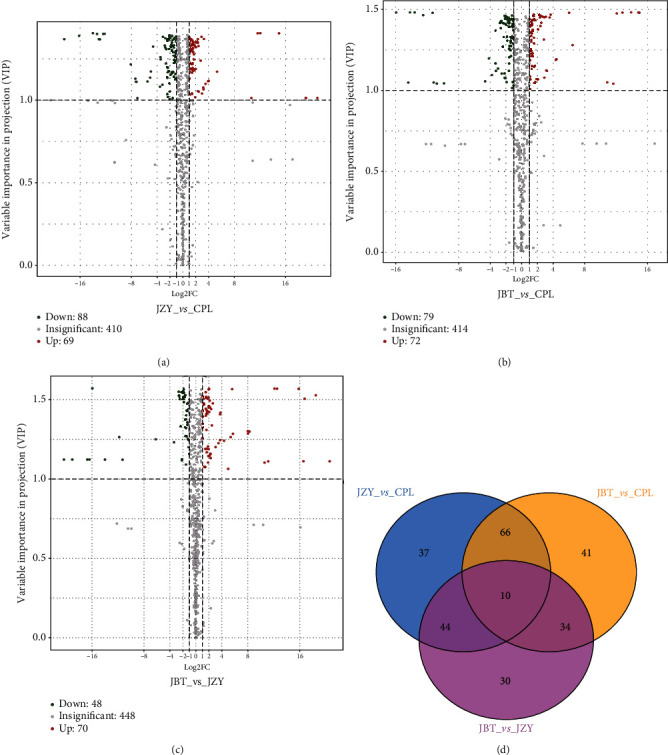
Differential metabolites in pairwise comparison among the sweet corn cultivars: (a) volcanic plots of differential metabolites in JZY vs. CPL; (b) volcanic plots of differential metabolites in JBT vs. CPL; (c) volcanic plots of differential metabolites in JBT vs. JZY; (d) overlap of differentially expressed metabolites in sweet corn kernels (VIP > 1). The number in each subcollection refers to quantity of metabolites in intersection.

In total, 262 metabolites were screened with significant differential amounts among cultivars by statistical analysis (Table S1). The number of differential metabolites (VIP > 1) was shown in the volcanic plots and Venn diagram (Figure 3). Compared with CPL, 151 and 157 metabolites were significantly changed in JBT and JZY, respectively (Figures 3(a) and 3(b)). These include 79 downregulated and 72 upregulated in JBT and 88 downregulated and 69 upregulated in JZY. There were 118 significantly changed metabolites in JBT vs. JZY, of which 48 were downregulated and 70 were upregulated in JBT (Figure 3(c)). The maximum variation was identified between high-quality cultivar CPL and medium-quality cultivar JZY. Among 157 significantly different metabolites, 88 of them with higher accumulation in medium-quality cultivar JZY were mainly classified into flavonoids and phenylpropanoids while CPL produced relatively more amino acids compared to JZY.

### 3.5. Metabolic Pathway Analysis Based on the KEGG Classification of Differential Metabolism

**Figure 4:**
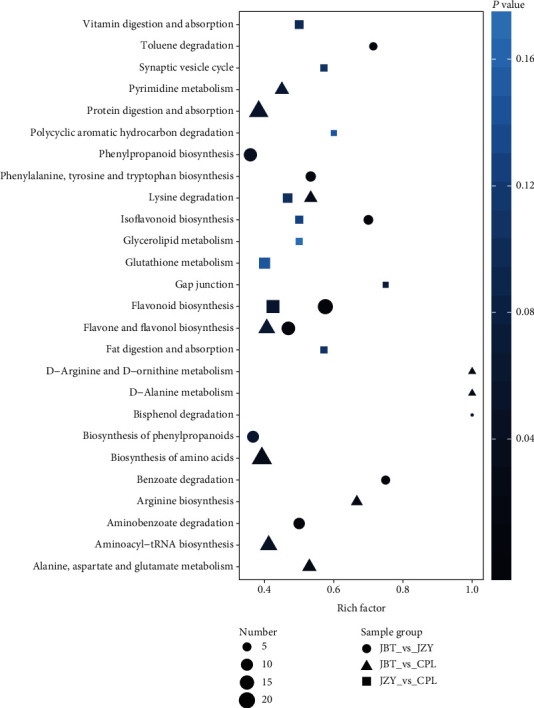
The enriched KEGG pathway terms covered by differential metabolites among three sweet corn cultivars. Rich factor is the percentage of the differential metabolite numbers in the corresponding pathway to the total annotated metabolite numbers in this pathway. The dot refers to the JBT_vs_JZY, the triangle represents the JBT_vs_CPL, and the square appoints to the JZY_vs_CPL. The size of the dots indicates the number of differential metabolites in the corresponding pathway.

The KEGG pathway classification was performed in the pairwise comparison of the three sweet corn cultivars. The ten most enriched pathway terms in pairwise comparison were combined in one KEGG enrichment diagram (Figure 4). The differential metabolites in the JBT_vs_JZY group were annotated into flavonoid biosynthesis, flavone and flavonol biosynthesis, phenylpropanoid biosynthesis, benzoate degradation, and toluene degradation. The majority of differential metabolic pathways in the JBT_vs_CPL group were flavone and flavonol biosynthesis, biosynthesis of amino acids, protein digestion, and absorption. There was a lower amount of differential amino acids, flavone, and flavonol in CPL than that in JBT. The differential metabolites in the JZY_vs_CPL group were mainly mapped into flavonoid biosynthesis, glutathione metabolism, gap junction, lysine degradation, vitamin digestion, and absorption. Compared to JZY, CPL showed a lower accumulation of flavonoids, indicating the metabolic variation in different cultivars of sweet corn kernels.

### 3.6. Key Quality-Related Metabolites in the Sweet Corn's Kernels

**Figure 5:**
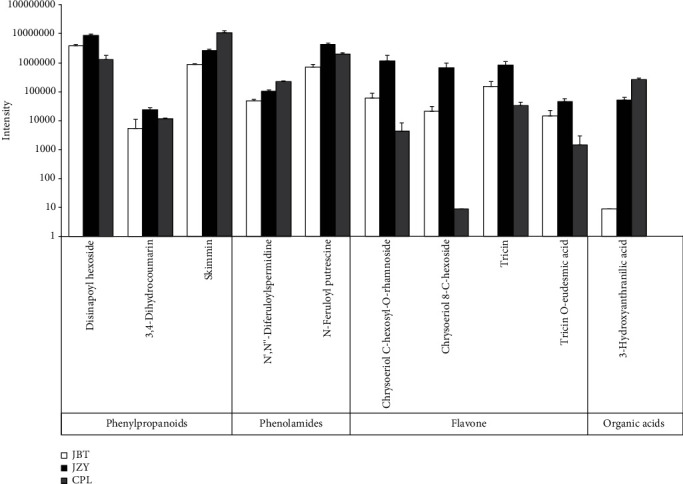
Ten significantly changed metabolites among all cultivars in the sweet corn's kernels.

We focused on the ten metabolites showing significant differences among all cultivars (Figure 3(d)). These significant metabolites respond for discrimination and contain phenylpropanoids, phenolamides, flavone, and organic acid (Figure 5). Of them, disinapoyl hexoside, 3,4-dihydrocoumarin, N-feruloyl putrescine, chrysoeriol C-hexosyl-O-rhamnoside, chrysoeriol 8-C-hexoside, tricin, and tricin O-eudesmic acid showed the highest accumulation in the medium-quality cultivar, JZY. It is consistent with the highest accumulation of phenylpropanoids, phenolamides, and flavonoids in JZY (Figure 2). However, skimmin and N′,N^″^-diferuloylspermidine were exceptions to the detected phenylpropanoids and phenolamides, respectively, in JZY. Skimmin and N′,N^″^-diferuloylspermidine showed the highest accumulation in the high-quality cultivar (CPL) and lowest intensities in low-quality cultivar, JBT. In addition, 3-hydroxyanthranilic acid displayed the same trend with skimmin and N′,N^″^-diferuloylspermidine. Most importantly, we found that skimmin, N′,N^″^-diferuloylspermidine, and 3-hydroxyanthranilic acid showed higher VIP values (VIP ≥ 1.3) than the rest of the seven metabolites (Table 1, Figure S4); thus, we identify these metabolites as the key quality-related metabolites of kernels for the high-quality sweet corn.

## 4. Discussion

Due to the technological development in the metabolomic field, the occurrence of quality breeding to the nutrient constituents of cereal crops is becoming attractive. The metabolomic analysis has been observed in many cereals including rice [9, 17, 18], wheat [19], and maize [7, 8]. However, knowledge of sweet corn kernel metabolome is either lacking or rare. The present work provides the comprehensive metabolism profiling of sweet corn kernels and provides an insight into the grain nutrient of sweet corn through the HPLC-MS-based metabolome techniques. A total of 262 differential metabolites were detected from three widely cultivated sweet corn cultivars. These metabolites were mapped into primary metabolic pathways, such as organic acids and amino acids, and secondary metabolic pathways, including phenylpropanoid and flavonoid pathways. Although previous studies also detected organic acids and amino acids in sweet corn kernels by GC-MS [11], few mentioned secondary metabolites, such as phenylpropanoids and flavonoids in sweet corn kernels.

Free amino acids are essential nutrition from corn kernel for human diet [20]. The free amino acid profiles were different for all studied three cultivars (Table 1). DL-methionine was the most abundant amino acid in all three cultivars whereas CPL was abundant in L-threonine, while JBT and JZY were abundant in L-(+)-arginine and L-asparagine, respectively. Of them, methionine and threonine are the essential amino acids for the human body. However, researchers found that the most abundant amino acids are glutamic acid, glutamine, leucine, and alanine in field maize kernels [21]. These free amino acids were detected abundantly in sweet corn as well (Table S1).

Organic acids were the second abundant primary metabolites in sweet corn kernels (Figure 2). Results showed that in addition to citric acid cycle, organic acids of sweet corn kernels were mainly concentrated on shikimate, benzoic acids, and quinic acid pathways (Table 1). The previous studies have shown that organic acids are mainly present in bound form in sweet corn kernels [20], which is consistent with our results. In the high-quality sweet corn cultivar CPL, quinic and shikimic acids were accumulated in O-glucoside form, while benzoic acid was accumulated in aldehyde form (Table 1). Many aromatic groups have been detected as modified quinic acid, like caffeoyl, coumaroyl, feruloyl, and sinapoyl in JBT and JZY (Table 1). Furthermore, the results have shown that CPL showed relatively high accumulation of kynurenic acid, fumaric acid, 2-hydroxyisocaproic acid, and acetic acid (p-hydroxyphenyl), while JBT had relatively large amounts of suberic acid, 2-methylglutaric acid, gallic acid O-hexoside, and Dl-2-aminooctanoic acid (Table 1). In brief, we found that the organic acid profiles in the three cultivars were diverse, which may contribute to the quality-related difference among these cultivars.

Two important categories of secondary metabolites, terpenes and alkaloids, were also detected in the present study. The most abundant differential alkaloid was trigonelline (Table 1), which is beneficial in the prevention and treatment of diabetes and central nervous system diseases [22, 23]. The biological significance of these alkaloids in sweet corn kernels needs to be further investigated in the future. In addition, the differential terpenes included phytocassane D, saikosaponin D, oleanolic acid, sweroside, ginkgolide B, and gentiopicroside among three sweet corn kernels (Table 1). However, terpenes were detected with low intensity in sweet corn kernels. It might be that the content of terpenes is low in sweet corn kernels, or the detection program, such as elution gradient, should be improved for terpenes' detection.

Secondary metabolites, such as phenolics, may provide health benefits strongly associated with a reduced risk of developing chronic diseases [24]. We found that the amount and the chemical diversity of flavonoids were highest in JZY. CPL showed a lower accumulation of flavonoids than JBT and JZY (Figure 4, Table 1), indicating the metabolic variation in different cultivars of sweet corn kernels. The previous study showed that phenolics and flavonoids in sweet corn mainly exist in free form compared with the conventional corn [21], but we determined the high intensity of glycosylated phenolics. The most abundant differential polyphenols were protocatechuic acid O-glucoside (Table 1), which have shown the antioxidant activity [25]. In addition, coumarins, originated from the phenylpropanoid pathway, were abundant in sweet corn kernels. They were skimmin, O-feruloyl 4-hydroxylcoumarin, and N-sinapoyl hydroxycoumarin (Table 1). Coumarins have been used as nutraceuticals, food supplements, which is due to their biochemical and pharmacological activities, such as anticancer, antioxidant, anti-inflammation, anti-HIV, anticoagulant, antibacterial, analgesic, and comparative immune modulation [26–28]. In addition, coumarins have a sweet odor, like the scent of new-mown hay, which may contribute to the sweet corn kernel flavor.

JZY and JBT showed the similar soluble sugar content, but the pericarp thickness of JBT was significantly higher than JZY. The activities for two enzymes, C4H and CAD in phenylpropanoid and lignin pathway, were higher in JBT than that in JZY (Figure 1(f)). These data indicated the enhanced lignin metabolic flux and potential lignin accumulation in JBT, which might contribute to the thick pericarp of JBT kernels (Figure 1(d)). This phenome is in contrast with the accumulation of flavonoids in JZY and JBT, and JBT showed less flavonoid accumulation compared to JZY (Figure 2, TableS2). It might be due that the phenylpropanoid pathway moves to the lignin accumulation to enhance the pericarp thickness compared to the flavonoid metabolic flux, resulting in the weak flavonoid metabolic flux in JBT. The weak CHI enzyme activity in JBT confirmed that this cultivar showed weak flavonoid metabolic flux compared to the JZY (Figure 1(f)).

The high-quality sweet corn cultivar, CPL, showed a low flavonoid accumulation in comparison to JBT and JZY (Figure 2, Table S2), which is the same with the lowest pericarp thickness phenotype (Figure 1). These results are consistent with the low C4H, CAD, and CHI activity in CPL (Figure 1(f)), revealing the weak phenylpropanoids and flavonoid metabolic flux in CPL kernels. Furthermore, the high soluble sugar content, including sucrose, glucose, and fructose, indicated the low glycolysis process in CPL. Due to that, both phenylpropanoid and flavonoid pathways derive from the glycolysis; the low phenylpropanoid and flavonoid accumulation might be caused by the low glycolysis pathway in CPL.

In addition to genetic background, multiple factors including cultivated condition, input system [29], and stress environment [30, 31] may affect sweet corn metabolome [32, 33]. For example, sweet corn is sensitive to the temperature; researchers have shown that ascorbic acid and folate biosynthesis varied in sweet corn seedlings under high-temperature stress [34]. With global climate warming, it would be necessary to explore the variation of kernels under high-temperature stress. In this study, sweet corn kernels from one geographic location were used, which skipped the factors such as soil, microorganisms, and microclimatic conditions in different locations. In the future, the widely targeted metabolomics can also be considered to investigate the metabolic variations in the different stages of kernel development, in a specific environment, or in different geographic locations. Additionally, the transcriptomics data can be combined to elucidate the metabolic network of sweet corn kernels, which would provide metabolic network information for breeding the sweet corn with abundant nutrition.

## 5. Conclusions

In conclusion, the widely targeted metabolic profiling in three sweet corn cultivars with the difference in grain quality was investigated through HPLC-MS/MS analysis. PCA and HCA of the metabolic dataset showed clear distinctions among different cultivars. Furthermore, the VIP plot in the OPLS-DA model unveiled the key quality-related metabolites for high-quality sweet corn. Information in this work provides an insight into the kernel nutrient of sweet corn through the metabolomic techniques. The results suggested the variation of metabolic composition among three cultivars, providing the reference metabolic profiling for sweet corn kernels. In addition, skimmin, N′,N^″^-diferuloylspermidine, and 3-hydroxyanthranilic acid were identified as the key quality-related metabolites related to grain quality traits in sweet corn. The kernel metabolites and nutrient compositions might be an important potential factor for future sweet corn breeding.

## Figures and Tables

**Table 1:** VIP values for the ten differential metabolites in sweet corn kernels.

Classification	Differential metabolites	JBT_vs_JZY	JBT_vs_CPL	JZY_vs_CPL
Phenylpropanoids	Disinapoyl hexoside	1.47	1.25	1.32
Phenylpropanoids	3,4-Dihydrocoumarin	1.15	1.01	1.22
Phenylpropanoids	Skimmin	1.52	1.47	1.35
Phenolamides	N′,N^″^-Diferuloylspermidine	1.5	1.46	1.35
Phenolamides	N-Feruloyl putrescine	1.48	1.29	1.27
Flavone	Chrysoeriol C-hexosyl-O-rhamnoside	1.24	1.09	1.22
Flavone	Chrysoeriol 8-C-hexoside	1.06	1.05	1.39
Flavone	Tricin	1.34	1.1	1.32
Flavone	Tricin O-eudesmic acid	1.17	1.12	1.17
Organic acid	3-Hydroxyanthranilic acid	1.57	1.48	1.35

## Data Availability

The metabolomics data used to support the findings of this study are included within the supplementary information files.

## Supplementary Materials

Supplementary Materials Figure S1: metabolite distribution by PCA analysis in sweet corn cultivars. A clear distinction can be observed among the three cultivars. The three dots in each group are representative of the pooled samples performed for this study. In total, 30 biological replications were performed per cultivar, every ten of which were pooled to give one pooled sample. Mix is the mixture of JZY, JBT, and CPL. Figure S2: HCA analysis of sweet corn kernels from the three accessions. Figure S3: OPLS-DA analysis of sweet corn kernels from the three accessions in pairs. (A) OPLS-DA model; (B) OPLS-DA score plot; (C) OPLS-DA S-plot, red dots indicate the distinctive compounds (VIP ≥ 1); (D) OPLS-DA permutation test (*n* = 200), the horizontal lines indicate *R*^2^*Y* and *Q*^2^ in the original model, and red dot and blue dot represent *R*^2^*Y*′ and *Q*^2′^ of the model after *Y* replacement, respectively. All *R*^2^*Y*′ dots are below the original *R*^2^*Y* line, and all Q^2′^ dots are below the original *Q*^2^ line, indicating the OPLS-DA model is valid and suitable. Figure S4: mass spectrum of key metabolites related to grain quality traits in sweet corn. Table S1: the gradient program for HPLC analysis in various sweet corn kernels. Table S2: a list of differential metabolites in kernels among the three sweet corn cultivars using UPLC-QTRAP-MS/MS (*Q*^2^ > 0.5 in OPLS-DA plot).
