# Supplementary material for: Widely Targeted Metabolomics Analysis Reveals Key Quality-Related Metabolites in Kernels of Sweet Corn
Source: Int J Genomics. 2021 Feb 8;2021:2654546. doi: 10.1155/2021/2654546 (PMC7884158; doi:10.1155/2021/2654546)
Supplement: Supplementary Materials — Figure S1: metabolite distribution by PCA analysis in sweet corn cultivars. A clear distinction can be observed among the three cultivars. The three dots in each group are representative of the pooled samples performed for this study. In total, 30 biological replications were performed per cultivar, every ten of which were pooled to give one pooled sample. Mix is the mixture of JZY, JBT, and CPL. Figure S2: HCA analysis of sweet corn kernels from the three accessions. Figure S3: OPLS-DA analysis of sweet corn kernels from the three accessions in pairs. (A) OPLS-DA model; (B) OPLS-DA score plot; (C) OPLS-DA S-plot, red dots indicate the distinctive compounds (VIP ≥ 1); (D) OPLS-DA permutation test (n = 200), the horizontal lines indicate R2Y and Q2 in the original model, and red dot and blue dot represent R2Y′ and Q2′ of the model after Y replacement, respectively. All R2Y′ dots are below the original R2Y line, and all Q2′ dots are below the original Q2 line, indicating the OPLS-DA model is valid and suitable. Figure S4: mass spectrum of key metabolites related to grain quality traits in sweet corn. Table S1: the gradient program for HPLC analysis in various sweet corn kernels. Table S2: a list of differential metabolites in kernels among the three sweet corn cultivars using UPLC-QTRAP-MS/MS (Q2 > 0.5 in OPLS-DA plot). [file 2654546.f1.pdf]

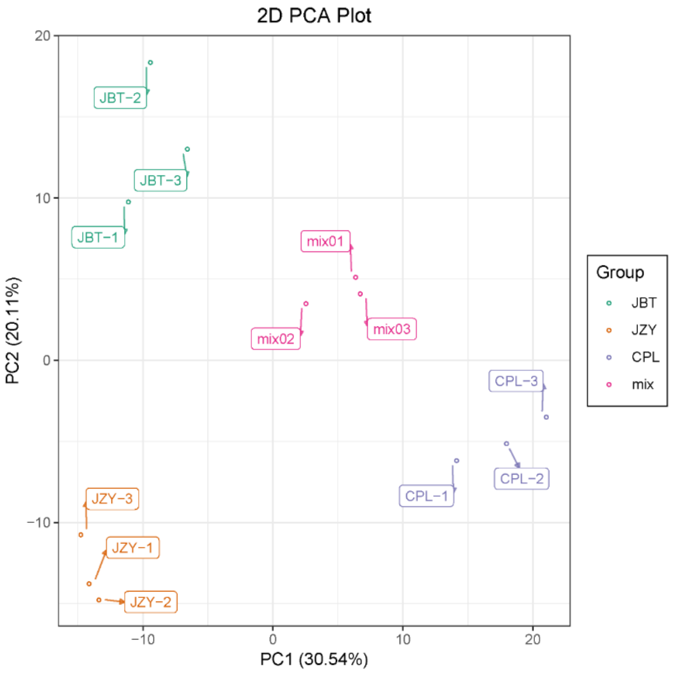

Figure S1: Metabolite distribution by PCA analysis in sweet corn cultivars. A clear distinction can be observed among the three cultivars. The three dots in each group are representative of the pooled samples performed for this study. In total, 30 biological replications were performed per cultivar, every ten of which were pooled to give one pooled sample. Mix is the mixture of JZY, JBT and CPL.

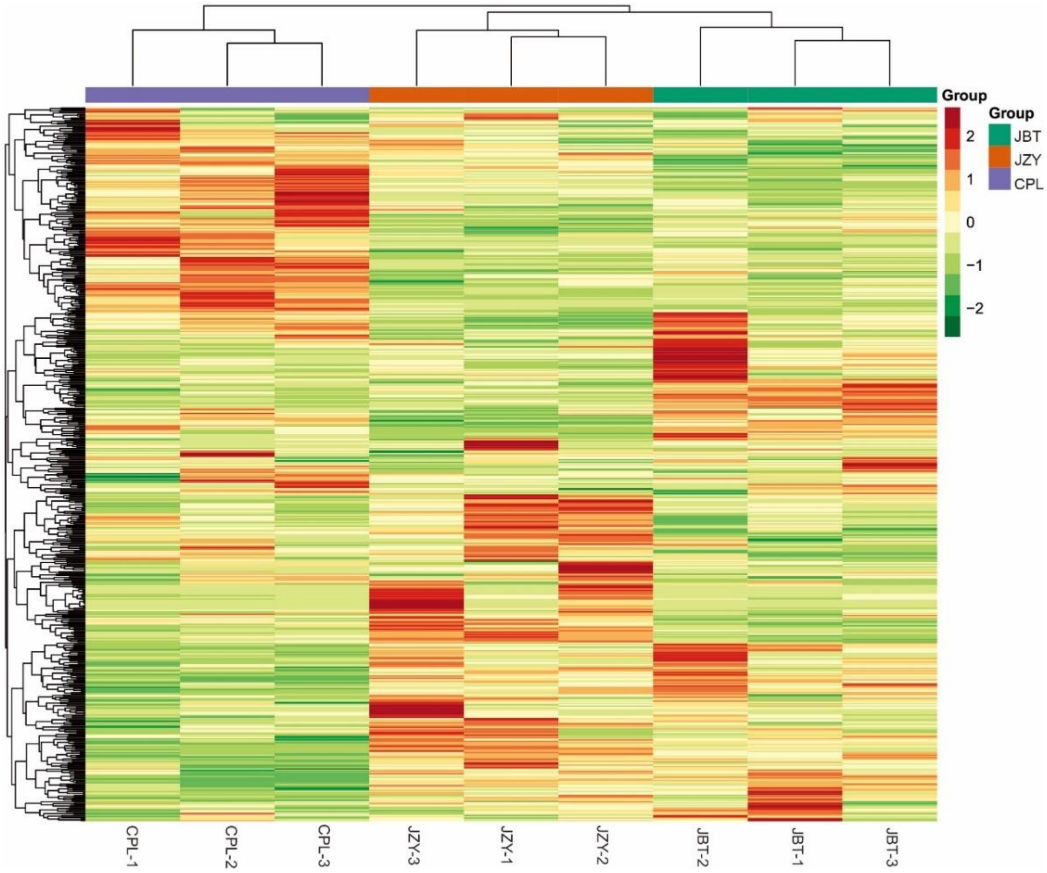

Figure S2. HCA analysis of sweetcorn kernels from the three accessions.

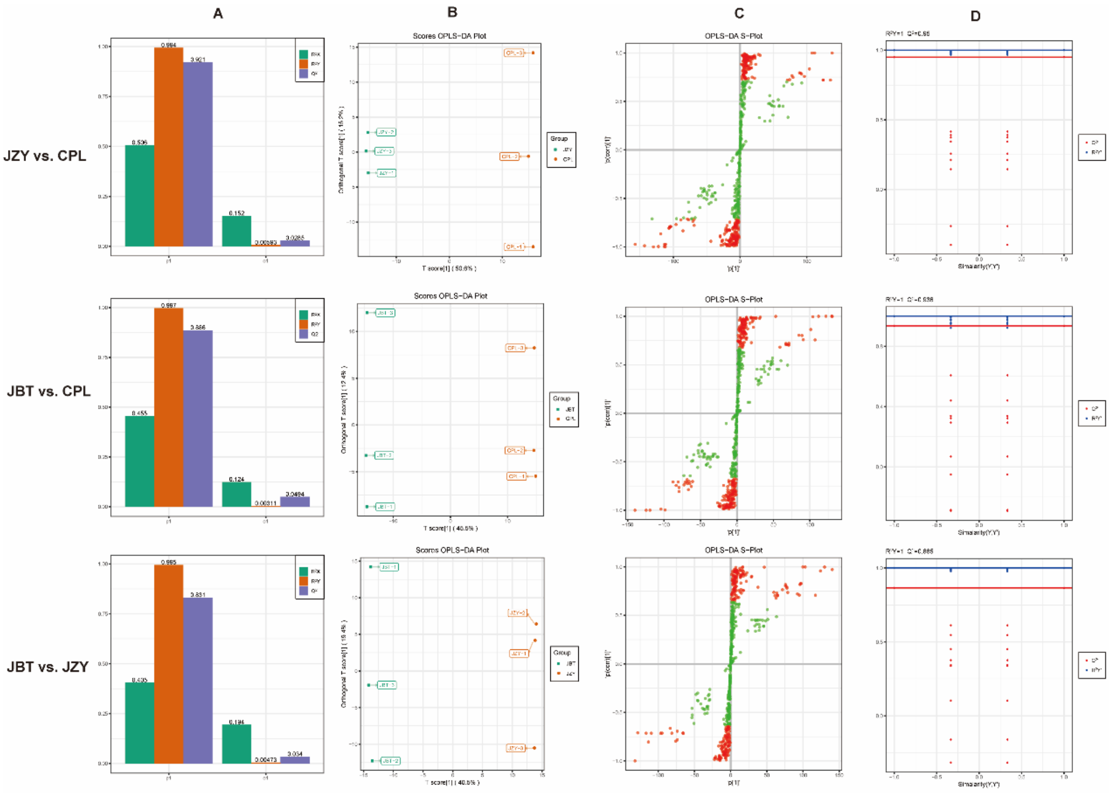

Figure S3. OPLS-DA analysis of sweetcorn kernels from the three accessions in pairs. (A), OPLS-DA model; (B), OPLS-DA scores plot, the three dots in each group are representative of the pooled samples performed for this study. In total, 30 biological replications were performed per cultivar, every ten of which were pooled to give one pooled sample; (C), OPLS-DA S-plot, red dots indicate the distinctive compounds (VIP≥1); (D), OPLS-DA permutation test (n=200), the horizontal lines indicate R^2Y and Q^2 in the original model, red dot and blue dot represent R^2Y' and $Q^{2\prime}$ of the model after Y replacement, respectively. All R^2Y' dots are below the original R^2Y line, and all $Q^{2\prime}$ dots are below the original Q^2 line, indicating the OPLS-DA model is validate and suitable.

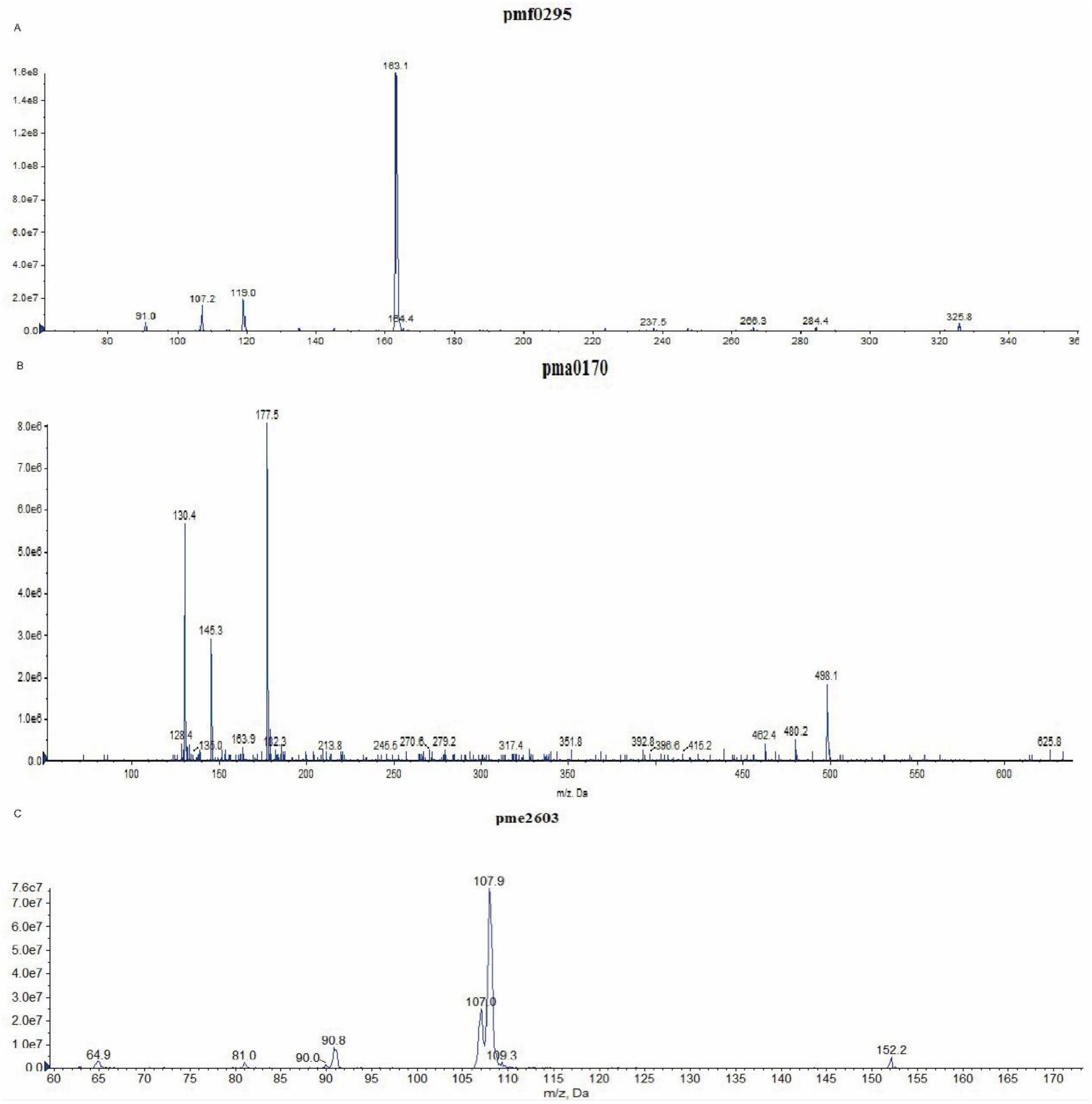

Figure S4. Mass spectrum of potential metabolite markers related to grain quality traits in sweet corn. (A), skimmin; (B), N′,N′′-diferuloylspermidine; (C), 3-hydroxyanthranilic acid.

Table S1. The gradient program for HPLC analysis in various sweet corn kernels.

	Time (min)	Flow rate (mL/min)	% A	% B
Gradient program	0.0	0.4	95	5
	11.0	0.4	5	95
	12.0	0.4	5	95
	12.1	0.4	95	5
	15.0	0.4	95	5

Table S2. A list of differential metabolites in kernels among the three sweet corn cultivars using UPLC-QTRAP-MS/MS ($Q^2 > 0.5$ in PLS-DA plot).

Class	Rt (min)	Q1 (Da)	Q3 (Da)	Molecular Weight (Da)	Ionization model	Compounds	JBT vs. JZY		JBT vs. CPL		JZY vs. CPL	
							VIP [a]	Log2FC [b]	VIP	Log2FC	VIP	Log2FC
Alcohols	0.76	204	102	205	[M-H]-	Pantothenol	1.16	-0.71	1.31	-1.35	1.14	-0.64
	0.77	151	71	152	[M-H]-	D-Arabitol	0.4	0.23	1.23	1.14	1.12	0.91
	0.77	169	134	168	[M+H]+	4-(Aminomethyl)-5-(hydroxymethyl)-2-methylpyridin-3-ol	1.4	-0.59	1.42	-1.59	1.25	-1
Alkaloids	0.79	138	94	137	[M+H]+	Trigonelline	1.52	-2.58	0.49	-0.247	1.37	2.33
	2.68	219	146	218	[M+H]+	Abrine	1.05	-0.43	1.37	-1.04	1.32	-0.61
	2.73	250	147	250	Protonated	Coumaroyl choline	1.55	-2.66	1.09	-0.929	1.18	1.73
	3.08	310	251	310	Protonated	Sinapoylcholine	1.56	1.96	0.315	0.192	1.32	-1.77
	3.39	157	103	156	[M+H]+	2,3'-Bipyridine	0.86	0.62	1.4	2.38	1.25	1.76
	3.79	485	323	484	[M+H]+	N-hexosyl-p-coumaroyl serotonin	0.55	-0.24	1.48	-13.6	1.4	-13.36
	3.84	219	174	218	[M+H]+	5-Methoxy-N,N-dimethyltryptamine	1.12	-11.3	0.0434	-0.621	1.01	10.68
	4.38	162	116	161	[M+H]+	Indole-3-carboxylic	1.49	2.13	1.42	2.5	0.63	0.37
	4.50	164	149	165	[M-H]-	Coixol	0.73	-0.83	1.12	-1.62	1.11	-0.78
	6.09	327	171	350	[M]-	Acid orange 20	1.2	0.75	0.869	-0.587	1.37	-1.34
Amino acid and derivatives	0.73	175	116	174	[M+H]+	L-(+)-Arginine	0.74	-0.14	1.4	-1.92	1.32	-1.78
	0.75	116	74	117	[M-H]-	Guanidineacetic acid	1.05	-0.67	1.46	-2.03	1.14	-1.36
	0.75	159	88	160	[M-H]-	D-Alanyl-D-Alanine	0.85	-0.37	1.44	-1.52	1.23	-1.15
	0.76	118	74	119	[M-H]-	L-Threonine	0.84	0.48	1.36	1.09	1.08	0.61
	0.76	147	84	146	[M+H]+	L-Glutamine	1.12	0.16	1.39	1.02	1.3	0.86
	0.77	307	145	308	[M-H]-	L-Glutamine O-hexside	0.84	-0.72	1.28	-1.52	0.99	-0.8
	0.77	122	76	121	[M+H]+	L-Cysteine	0.74	0.2	1.24	1.22	1.13	1.01
	0.77	399	250	398	[M+H]+	S-(5'-Adenosyl)-L-methionine	1.12	-2.08	0.67	-0.712	0.47	1.37
	0.78	147	84	146	[M+H]+	L-(+)-Lysine	1.13	0.17	1.44	1	1.37	0.83
	0.78	131	114	132	[M-H]-	L-Asparagine	1.39	-0.74	1.42	-1.62	1.29	-0.88
	0.78	133	116	132	[M+H]+	L(+)-Ornithine	1.03	-0.82	1.27	-1.33	0.88	-0.51
	0.78	88	71	89	[M-H]-	L-Alanine	1.48	-0.39	1.44	-1.03	1.33	-0.64

	0.79	174	131	175	[M-H]-	L-Citrulline	0.89	-0.25	1.41	-1.11	1.19	-0.86
	0.79	275	257	276	[M-H]-	L-Saccharopine	1.5	0.86	1.45	3.07	1.35	2.21
	0.80	162	98	161	[M+H]+	2-Aminoadipic acid (L-Homoglutamic acid)	0.68	0.28	1.45	3.56	1.37	3.28
	0.80	235	118	234	[M+H]+	Lysine butyrate	1.5	-1.9	1.27	-1.49	0.48	0.41
	0.80	308	146	309	[M-H]-	L-Glutamic acid O-glucoside	1.27	-1.4	0.64	0.339	1.28	1.73
	0.84	112	95	111	[M+H]+	Histamine	0.53	-0.31	1.15	1.55	1.19	1.87
	0.87	150	61	149	[M+H]+	DL-Methionine	1.56	0.88	1.17	-0.353	1.37	-1.23
	1.11	187	125	188	[M-H]-	Nα-Acetyl-L-glutamine	0.735089	-0.80328	1.135235	-3.01054	0.523951	-2.20726
	1.13	251	84	250	[M+H]+	γ-Glu-Cys	0.321016	0.076869	1.310139	1.198614	1.319098	1.121745
	1.14	187	145	188	[M-H]-	N6-Acetyl-L-lysine	1.47	-0.99	1.1	0.269	1.36	1.26
	1.14	217	158	216	[M+H]+	Nα-Acetyl-L-arginine	1.463957	-0.9678	1.399219	-1.17645	0.764004	-0.20866
	1.15	150	61	149	[M+H]+	L-Methionine	1.37	0.67	1.26	-0.41	1.37	-1.08
	1.16	308	233	307	[M+H]+	Glutathione reduced form	1	0.4	1.25	1.13	1.05	0.74
	1.16	128	82	129	[M-H]-	L-Pipecolic acid	0.82	-0.3	1.39	-1.05	1.23	-0.75
	1.16	217	199	218	[M-H]-	(5-L-Glutamyl)-L-amino acid	1.09	-1.44	0.824	-0.527	0.95	0.91
	1.18	188	128	189	[M-H]-	N-Acetyl-L-glutamic acid	0.22	0.06	1.39	-1.6	1.32	-1.66
	1.26	132	86	131	[M+H]+	L-Isoleucine	1.02	-0.24	1.41	0.785	1.36	1.02
	1.26	138	103	137	[M+H]+	L-Tyramine	1.31	-0.93	1.32	1.5	1.33	2.43
	1.27	174	88	175	[M-H]-	N-Acetylaspartate	0.69	-10.46	1.04	1.6	1.41	12.06
	1.29	132	57	131	[M+H]+	N-Propionylglycine	1.09	-0.23	1.4	0.799	1.38	1.02
	1.35	132	86	131	[M+H]+	alpha-Aminocaproic acid	1.01	-0.27	1.4	0.757	1.36	1.02
	1.44	162	73	161	[M+H]+	S-Allyl-L-cysteine	0.8	2.96	1.1	-2.53	1.11	-5.49
	1.45	212	166	211	[M+H]+	(-)-3-(3,4-Dihydroxyphenyl)-2-methylalanine	0.8	0.32	1.05	-1.14	1.17	-1.47
	1.50	210	135	211	[M-H]-	3-(6-Hydroxy-3,4-dioxo-1,5-cyclohexadien-1-yl)-L-alanine	1.19	-0.41	1.46	-1.24	1.27	-0.83
	1.59	161	99	162	[M-H]-	3-Hydroxy-3-methylpentane-1,5-dioic acid	1.18	-0.59	1.15	0.623	1.36	1.21
	1.91	216	170	215	[M+H]+	3-Chloro-L-tyrosine	1.12	-14	0.457	-0.291	0.64	13.71

	1.92	166	77	165	[M+H]+	D-(+)-Phenylalanine	1.12	-19.04	0.0141	0.0969	1.01	19.14
	1.98	237	192	236	[M+H]+	N'-Formylkynurenine	1.12	-2.06	0.956	-0.756	0.06	1.31
	2.57	145	101	146	[M-H]-	2,3-dimethylsuccinic acid	1.48	-1.78	1.42	-2.69	0.96	-0.91
	2.61	224	136	223	[M+H]+	N-Acetyl-L-tyrosine	1.25	-1.18	1.32	-1.29	0.06	-0.12
	3.51	172	130	173	[M-H]-	N-Acetyl-l-leucine	1.4	0.78	1.46	2.16	1.33	1.38
	3.62	313	166	312	[M+H]+	Phe-Phe	1.41	1.75	0.37	0.11	1.29	-1.64
	3.84	206	147	207	[M-H]-	N-Acetyl-L-phenylalanine	1.25	3.74	0.596	2.85	0.67	-0.88
	4.07	245	88	246	[M-H]-	N-(3-Indolylacetyl)-L-alanine	0.69	-9.93	1.05	1.72	1.41	11.65
	7.49	302	109	301	[M+H]+	D-erythro-sphinganine	1.52	1.05	0.787	0.348	1.19	-0.7
Carbohydrates	0.76	649	145	650	[M-H]-	D(+)-Melezitose O-rhamnoside	0.97	-0.43	1.33	-1.39	1.12	-0.96
	1.07	289	97	290	[M-H]-	D-Sedoheptuiose 7-phosphate	0.22	0.05	1.31	2.82	1.24	2.77
Flavonoids and proanthocyanins	4.07	609	301	610	[M-H]-	Hesperetin 7-O-neohesperidoside (Neohesperidin)	1.44	2.35	0.291	-0.38	1.37	-2.73
	4.08	609	301	610	[M-H]-	Hesperetin 7-rutinoside (Hesperidin)	1.45	2.14	0.522	-0.451	1.31	-2.59
	4.22	433	271	434	[M-H]-	Naringenin 7-O-glucoside (Prunin)	1.53	18.46	- [d]	-	1.37	-18.46
	5.05	289	153	288	[M+H]+	Eriodictyol	0.28	-0.12	1.46	-12.5	1.4	-12.39
	5.57	271	151	272	[M-H]-	Naringenin chalcone	1.3	8.27	0.491	1.2	1.01	-7.07
	5.59	271	151	272	[M-H]-	Naringenin [C]	1.51	16.74	0.671	9.52	1.11	-7.23
	5.59	271	151	272	[M-H]-	Butin	1.3	7.98	0.0322	0.604	1.13	-7.38
	5.66	271	151	272	[M-H]-	4',5,7-Trihydroxyflavanone	1.29	8.02	0.0373	0.705	1.11	-7.31
	5.75	301.1	164	302	[M-H]-	Hesperetin	1.26	5.35	0.669	-7.71	1.37	-13.07
	5.75	301.1	151	302	[M-H]-	Homoeriodictyol	1.28	5.71	0.669	-7.2	1.37	-12.91
	2.63	487	163	488	[M-H]-	Acacetin O-acetyl hexoside	1.47	1.09	1.27	0.582	1.05	-0.51
	2.78	627	465	626	[M+H]+	8-C-hexosyl-hesperetin O-hexoside	0.86	0.19	1.42	1.18	1.33	0.98
	2.83	771	609	772	[M-H]-	Luteolin O-hexosyl-O-hexosyl-O-hexoside	1.27	-1.51	0.174	0.0781	0.9	1.59
	3.39	565	433	564	[M+H]+	C-hexosyl-apigenin C-pentoside	1.19	1.98	0.038	-0.14	1.17	-2.12
	3.72	669	331	668	[M+H]+	Tricin 5-O-feruloylhexoside	1.46	2.03	0.872	-0.779	1.33	-2.81
	3.77	565	499	564	[M+H]+	C-pentosyl-C-hexosyl-apigenin	1.14	1.92	0.519	-0.481	1.14	-2.4
	3.78	433.1	283.1	432	[M+H]+	Apigenin C-glucoside	1.2	3.04	1.07	2.02	0.84	-1.02
	3.78	433.1	283	432	[M+H]+	Isovitexin	0.59	2.53	1.07	1.87	0.47	-0.66
	3.84	419	383	418	[M+H]+	O-methylnaringenin C-pentoside	1.24	-1.05	0.623	-0.313	0.67	0.74
	3.87	609	463	608	[M+H]+	Chrysoeriol C-hexosyl-O-rhamnoside	1.24	4.33	1.09	-3.79	1.22	-8.11

	3.89	463	313	462	[M+H]+	Chrysoeriol 8-C-hexoside	1.06	4.98	1.05	-11.2	1.39	-16.15
	3.94	503	341	504	[M-H]-	Chrysoeriol O-acetylhexoside	0.76	-0.53	1.08	-2.06	1.01	-1.53
	4.00	491	329	492	[M-H]-	Tricin 5-O-hexoside	0.7	0.53	0.786	1.64	1.28	1.11
	4.07	637	329	638	[M-H]-	Tricin 5-O-rutinoside	1.43	-1.36	0.931	0.691	1.32	2.05
	4.11	527	331	526	[M+H]+	Tricin 5-O-β-guaiacylglycerol	1.309515	1.621293	0.753975	0.750212	0.954408	-0.87108
	4.30	491	315	492	[M-H]-	Tricin 7-O-hexoside	0.97	0.86	1.48	-11.3	1.4	-12.19
	4.56	579	331	578	[M+H]+	Tricin O-malonylhexoside	0.09	1.1	1.04	-9.87	1	-10.97
	5.23	403	313	404	[M-H]-	Tricin O-glycerol	0.49	-0.14	1.17	-3.32	1.09	-3.18
	5.48	435	331	434	[M+H]+	Tricin O-phenylformic acid	1.1	10.57	1.04	11.6	0.08	1.08
	5.73	329	314	330	[M-H]-	Tricin	1.34	2.46	1.1	-2.18	1.32	-4.63
	5.76	299	284	300	[M-H]-	Chrysoeriol			1.05	10.9	0.99	10.87
	6.00	523	329	524	[M-H]-	Tricin O-eudesmic acid	1.19	1.69	1.12	-3.28	1.17	-4.97
	3.34	563	353	596	[M-H]-	Schaftoside	1.21	1.85	0.892	-0.79	1.22	-2.64
	3.50	563	353	564	[M-H]-	Vicenin-3	1.17	1.68	0.759	-0.676	1.2	-2.35
	3.52	565	409	564	[M+H]+	Isoschaftoside	1.08	1.45	0.808	-0.719	1.17	-2.17
	3.97	449	151	450	[M-H]-	Astilbin	1.11	16.57	0.671	7.72	0.76	-8.85
	4.00	465	301	464	[M-H]-	Gossypitrin	1.11	11.14	-	-	1	-11.14
	4.14	607	299	608	[M-H]-	Diosmin	1.39	1.07	1.07	1.1	0.09	0.04
	4.18	479	317	478	[M+H]+	Isorhamnetin 3-O-glucoside	1.57	15.82	0.671	10.7	1.14	-5.09
	4.47	179	137	178	[M+H]+	5,7-Dihydroxychromone	1.32	-1.45	1.33	-2.22	0.85	-0.77
	4.92	593	285	594	[M-H]-	Tiliroside	1.26	1	1.23	-0.653	1.3	-1.65
	5.13	303	121	302	[M+H]+	Herbacetin	1	0.37	1.32	-1.03	1.28	-1.4
	3.86	463	301	464	[M-H]-	Quercetin 4'-O-glucoside (Spiraeoside)	1.11	20.6	1.48	-15.9	1	-20.6
	3.86	447	285	448	[M-H]-	Kaempferol 3-O-galactoside (Trifolin)	1.23	-3.37	1.2	-4.16	0.04	-0.78
	3.36	417	255	416	[M+H]+	Daidzein 7-O-glucoside (Daidzin)	1.5	1	1.45	2.33	1.3	1.33
	3.54	445	283	446	[M-H]-	Glycitin	1.25	-6.19	1.06	-4.65	0.12	1.54
	2.31	155	109	154	[M+H]+	Protocatechuic acid	1.54	-1.73	0.97	-0.71	1.03	1.02
	2.44	315	153	316	[M-H]-	Protocatechuic acid O-glucoside	1.56	-1.79	0.639	-0.286	1.22	1.51
	3.03	139	93	138	[M+H]+	Protocatechuic aldehyde	1.54	-2.13	0.59	-0.199	1.32	1.93
	4.80	715	563	716	[M-H]-	Theaflavin	1.01	-0.47	1.26	1.16	1.29	1.63
	2.92	577	425	576	[M+H]+	Procyanidin A3	0.71	8.9	1.48	12.1	1.08	3.18
	2.61	447	285	448	[M-H]-	Cyanidin 3-O-glucoside (Kuromanin)	1.51	1.57	1.44	1.59	0.1	0.02

	2.68	449	287	448	[M]+	Cyanidin 3-O-galactoside	0.62	0.15	0.758	1.44	1.18	1.29
	2.92	493	331	493	Protonated	Malvidin 3-O-glucoside (Oenin)	0.71	0.7	0.574	-2.86	1.08	-3.56
	3.03	697	535	697	Protonated	Malvidin 3-acetyl-5-diglucoside	0.71	10.36	1.48	13.9	1.1	3.54
	3.54	287	213	287	Protonated	Cyanidin	1.51	-1.86	1.26	-1.6	0.09	0.26
Indole derivatives	2.85	127	81	126	[M+H]+	Imidazole-4-acetate	1.13	2.28	1.12	3.21	1.22	0.93
	3.70	176	91	175	[M+H]+	5-methoxyindole-3-carbaldehyde	1.43	1.04	1.21	0.686	1.05	-0.35
Lipids	6.73	351	147	350	[M+H]+	MAG (18:4) isomer1	1.12	0.8	1.01	-1.2	1	-2
	6.84	538	184	537	[M+H]+	LysoPC 19:0	1.46	1.98	1.02	0.627	1.31	-1.35
	7.51	424	227	425	[M-H]-	LysoPE 14:0 (2n isomer)	1.2	0.66	1.1	-0.688	1.32	-1.35
	7.54	309	209	310	[M-H]-	13-HpOTrE(r)	0.68	1.05	0.942	-0.933	1.05	-1.98
	7.73	468	184	467	[M+H]+	LysoPC 14:0 (2n isomer)	1.47	1.24	1.34	0.851	0.96	-0.39
	7.80	351	147	350	[M+H]+	MAG (18:4) isomer3	1.33	-2.25	1.27	-2.35	0.25	-0.1
	7.88	494	184	493	[M+H]+	LysoPC 16:1 (2n isomer)	1.51	1.95	1.38	2.03	0.06	0.08
	8.42	293	275	294	[M-H]-	9-HOTrE	0.51	-0.93	0.794	1.55	1.05	2.47
	8.52	277	93	276	[M+H]+	14,15-Dehydrocrepenynic acid	0.75	0.35	1.06	-0.779	1.28	-1.13
	8.55	494	184	493	[M+H]+	LysoPC 16:1	1.57	2.03	1.47	1.49	1.3	-0.55
	8.73	452.3	255.2	453	[M-H]-	LysoPE 16:0 (2n isomer)	0.19	-0.28	1.17	-1.59	1.22	-1.32
	8.74	452	255.3	453	[M-H]-	LysoPE 16:0	0.21	-0.25	1.2	-1.58	1.24	-1.33
	8.98	482	314	481	[M+H]+	LysoPE 18:0 (2n isomer)	1.2	0.38	1.23	-0.999	1.26	-1.38
	9.29	297	183	298	[M-H]-	1-Eicosanol	1.48	-1.13	1.33	-1.18	0.21	-0.06
	9.34	510	184	509	[M+H]+	LysoPC 17:0	1.43	0.47	1.3	-0.61	1.36	-1.08
	9.90	353	335	352	[M+H]+	MAG (18:3) isomer4	1.44	-0.9	0.45	0.196	1.22	1.1
	9.91	524	184	523	[M+H]+	LysoPC 18:0	0.47	-0.17	1.39	-1.18	1.26	-1.01
	10.10	353	261	352	[M+H]+	MAG (18:3) isomer1	1.48	-1.09	0.0233	0	1.3	1.09
	10.50	355	263	354	[M+H]+	MAG (18:2)	0.99	-0.94	0.471	0.47	1.04	1.41
	10.70	309	227	310	[M-H]-	cis-Gondoic acid	1.1	1.87	0.44	0.186	0.76	-1.68
Nucleotide and derivates	0.70	333	137	332	[M+H]+	2'-Deoxyinosine-5'-monophosphate	1.17	-0.89	1.37	1.39	1.36	2.27
	0.82	112	95	111	[M+H]+	Cytosine	0.65	0.22	1.22	1.31	1.19	1.09
	0.82	322	97	323	[M-H]-	Cytidine 5'-monophosphate (Cytidylic acid)	1.23	0.64	1.15	1.28	0.65	0.64

	1.16	298	166	297	[M+H]+	N2-methylguanosine	1.22	-0.77	0.759	0.332	1.31	1.1
	1.16	228	112	227	[M+H]+	Deoxycytidine	0.44	0.21	1.43	1.26	1.22	1.06
	1.17	332	136	331	[M+H]+	2'-Deoxyadenosine-5'-monophosphate	0.55	0.13	1.36	-0.922	1.39	-1.06
	1.19	242	109	243	[M-H]-	Cytidine	1.5	0.77	1.4	1.3	1.09	0.52
	1.19	151	151	152	[M-H]-	Xanthine	1.48	0.76	1.43	1.4	1.25	0.64
	1.23	113	70	112	[M+H]+	Uracil	0.22	-0.08	1.23	1.34	1.17	1.42
	1.24	243	110	244	[M-H]-	Uridine	0.24	0.01	1.09	1.08	1.17	1.07
	1.27	115	98	114	[M+H]+	Dihydrouracil	0.71	0.77	0.799	1.79	1.03	1.02
	1.32	150	133	151	[M-H]-	Guanine	1.39	-0.76	0.967	0.51	1.29	1.27
	1.33	346	152	345	[M+H]+	Guanosine 3',5'-cyclic monophosphate	1.47	-0.8	1.15	0.778	1.31	1.58
	1.67	253	137	252	[M+H]+	2'-Deoxyinosine	1.12	-16.73	0.158	-0.0554	0.97	16.67
	1.85	167	150	166	[M+H]+	7-Methylxanthine	1.57	-15.94	-	-	-	-
	1.88	252	136	251	[M+H]+	Deoxyadenosine	1.12	-20.33	0.0381	0.624	1.01	20.95
	1.92	167	121	166	[M+H]+	6-Methylmercaptopurine	1.26	-11.81	0.634	-0.953	0.63	10.86
	1.94	400	136	399	[M+H]+	Adenosine O-ribose	1.12	-16.38	0.167	2.79	1.01	19.17
	2.18	312	180	311	[M+H]+	2-(dimethylamino)guanosine	0.97	-0.4	1.3	1.2	1.34	1.6
	2.27	282	150	281	[M+H]+	1-Methyladenosine	0.68	-0.3	1.35	1.28	1.34	1.58
	2.90	296	134	297	[M-H]-	5'-Deoxy-5'-(methylthio)adenosine	1.26	-0.44	1.44	-1.21	1.24	-0.77
	4.36	336	148	335	[M+H]+	Riboprine	1.33	-1.6	0.522	0.188	1.33	1.79
Organic acids and derivatives	0.75	145	101	146	[M-H]-	Methylglutaric acid	0.68	0.08	1.42	-1.57	1.34	-1.64
	0.76	108	64	109	[M-H]-	2-Aminoethanesulfinic acid	0.39	0.1	1.3	-1.38	1.32	-1.47
	0.79	193	113	194	[M-H]-	D-Galacturonic acid	0.604475	0.270269	1.21965	-0.7713	1.127082	-1.04157
	0.84	144	102	145	[M-H]-	4-Guanidinobutyric acid	0.57	-0.18	1.45	-1.89	1.39	-1.7
	0.86	191	111	192	[M-H]-	Citric acid	0.52	0.26	1.08	-2.16	1.04	-2.42
	0.92	191	85	192	[M-H]-	Quinic acid	0.86	0.23	1.25	-0.786	1.29	-1.01
	1.03	147	87	148	[M-H]-	Citramalate	0.93	0.2	1.26	-1.02	1.24	-1.23
	1.13	89	71	90	[M-H]-	Oxalic acid	1.03316	-0.51315	1.383091	-2.58601	1.303833	-2.07286
	1.19	115	71	116	[M-H]-	Fumaric acid	1.01	0.25	1.21	1.02	1.05	0.77
	1.19	89	59	90	[M-H]-	3-Hydroxypropanoic acid	0.72	0.37	1.02	-0.65	1.26	-1.02
	1.29	103	59	104	[M-H]-	3-Hydroxybutyrate	0.79	0.17	1.41	-0.895	1.35	-1.06
	1.56	103	57	104	[M-H]-	2-Hydroxybutanoic acid	0.49	-0.17	1.44	-1.48	1.38	-1.31

	1.56	103	57	104	[M-H]-	α-Hydroxyisobutyric acid	0.260642	-0.10891	1.420853	-1.69068	1.356446	-1.58178
	2.02	331	313	332	[M-H]-	Gallic acid O-Hexoside	1.54	-1.94	0.723	-1.21	0.53	0.72
	2.10	499	163	500	[M-H]-	5-O-p-coumaroyl quinic acid O-hexoside	1.43	1.73	0.726	1.29	0.62	-0.44
	2.26	359	182	360	[M-H]-	Syringic acid O-glucoside	1.43	-1.23	1.33	-0.864	0.96	0.37
	2.28	341	207	340	[M+H]+	Sinapoyl malate	1.57	5.59	1.48	6.02	1.37	0.44
	2.30	474	327	473	[M+H]+	10-Formyl-THF	0.24	-0.08	1.46	-2.17	1.39	-2.1
	2.35	353	191	354	[M-H]-	Neochlorogenic acid (5-O-Caffeoylquinic acid)	1.33	1.05	0.859	-0.555	1.16	-1.6
	2.38	353.1	191.1	354	[M-H]-	1-O-Caffeoyl quinic acid	1.35	1.03	0.861	-0.551	1.17	-1.58
	2.42	153	109	154	[M-H]-	2,3-Dihydroxybenzoic acid	1.53	-1.57	1.37	-0.985	1.29	0.59
	2.50	499	163	500	[M-H]-	3-O-p-coumaroyl quinic acid O-hexoside	1.41	1.62	0.682	1.22	0.58	-0.4
	2.50	153	108	154	[M-H]-	2,5-dihydroxybenzoic acid (Gentisic acid)	1.16	-1.01	1.25	-1.42	0.85	-0.41
	2.58	145	101	146	[M-H]-	2-Methylglutaric acid	1.5	-1.81	1.43	-2.66	0.97	-0.85
	2.62	481	319	482	[M-H]-	5-O-p-coumaroyl shikimic acid O-hexoside	1.43	1.02	1.34	1.18	0.33	0.16
	2.64	188	144	189	[M-H]-	Kynurenic acid	0.77	0.49	1.43	1.29	1.05	0.79
	2.66	152	108	153	[M-H]-	3-Hydroxyanthranilic acid	1.57	12.49	1.48	14.8	1.35	2.33
	2.72	353	191	354	[M-H]-	Chlorogenic acid (3-O-Caffeoylquinic acid)	1.26	0.95	0.932	-0.653	1.16	-1.61
	2.75	175	115	176	[M-H]-	2-Isopropylmalate	0.75	0.34	1.44	-1.84	1.37	-2.18
	2.75	153	109	154	[M-H]-	2,4-Dihydroxybenzoic acid	1.52	-1.64	1.26	-0.986	1.13	0.65
	2.76	131	85	132	[M-H]-	5-hydroxyhexanoic acid	0.85	0.29	1.45	-1.67	1.38	-1.96
	2.85	337	173	338	[M-H]-	1-O-p-Coumaroyl quinic acid	1.19	1.47	0.913	-0.816	1.16	-2.29
	2.92	353	135	354	[M-H]-	Cryptochlorogenic acid	1.43	1.71	1.25	1.09	0.8	-0.62
	3.01	369	177	368	[M+H]+	3-O-Feruloyl quinic acid	1.37	-1.42	1.41	-2.05	0.66	-0.62
	3.10	137	93	138	[M-H]-	4-Hydroxybenzoic acid	1.55	-1.84	0.66	-0.33	1.19	1.51
	3.13	160	114	159	[M+H]+	Dl-2-Aminooctanoic acid	1.36	-1.24	1.31	-0.727	0.86	0.51
	3.13	515	191	516	[M-H]-	Cynarin	1.51	1.85	1.46	2.67	1.32	0.81
	3.14	137	94	136	[M+H]+	p-Aminobenzoate	0.87	-2.16	1.39	-1.54	0.65	0.62
	3.17	399	207	398	[M+H]+	O-Sinapoyl quinic acid	1.47	-1.26	1.44	-2.11	1.37	-0.85
	3.21	151	107	152	[M-H]-	p–Hydroxyphenyl acetic acid	-	-	1.48040	14.9691	1.40482	14.9691

									6	2	8	2
	3.23	481	319	482	[M-H]-	3-O-p-coumaroyl shikimic acid O-hexoside	1.18	0.74	1.19	1.06	0.71	0.32
	3.26	337	163	338	[M-H]-	5-O-p-Coumaroylquinic acid	1.08	1.31	0.846	-0.719	1.1	-2.02
	3.42	369	177	368	[M+H]+	1-O-Feruloyl quinic acid	1.5	-2.42	1.43	-2.56	0.19	-0.14
	3.52	131	85	132	[M-H]-	2-Hydroxyisocaproic acid	0.48	-0.33	1.04	0.938	1.21	1.27
	3.72	121	92	122	[M-H]-	4-Hydroxybenzaldehyde	1.55	1.53	1.46	1.48	0.31	-0.04
	3.74	369	163	368	[M+H]+	Chlorogenic acid methyl ester	0.04	0.39	1.19	4.41	1.12	4.03
	3.81	321	147	320	[M+H]+	5-O-p-Coumaroyl shikimic acid	0.02	0.36	1.05	-14.4	1	-14.77
	3.87	173	111	174	[M-H]-	Suberic acid	1.36	-0.9	1.44	-1.29	0.74	-0.38
	3.93	319	145	320	[M-H]-	3-O-p-Coumaroyl shikimic acid	0.1	-0.15	1.37	-3.24	1.38	-3.1
	3.95	195	121	196	[M-H]-	(3,4-Dimethoxyphenyl) acetic acid	0.04	-0.21	1.04	-10.8	0.98	-10.57
	3.98	517	163	516	[M+H]+	Isochlorogenic acid B	1.23	3.47	1.19	4.33	0.86	0.87
	4.00	151	136	152	[M-H]-	Vanillin	1.51	1.05	1.43	0.827	1.04	-0.23
	4.16	153	92	152	[M+H]+	2-Methoxybenzoic acid	1.32	-0.93	1.42	-2.73	1.32	-1.8
	4.62	211	137	212	[M-H]-	Eudesmic acid (3,4,5-trimethoxybenzoic acid)	1.4	-1.42	0.341	0.114	1.34	1.53
Others	0.79	106	88	105	[M+H]+	Diethanolamine	1.32	-0.2	1.48	-14.2	1.41	-14
	1.15	300	97	301	[M-H]-	N-Acetylglucosamine 1-phosphate	0.64	-0.36	1.15	1.64	1.19	2
	2.05	374	198	373	[M+H]+	DIMBOA glucoside	0.64	0.14	0.803	2.48	1.05	2.34
	3.79	503	311	502	[M+H]+	Phellodensin F	1.53	-2.62	1.31	-1.8	0.74	0.82
Phenolamides	0.56	146	72	145	[M+H]+	Spermidine	1.3	-0.97	1.22	0.979	1.35	1.95
	0.76	103	86	102	[M+H]+	1,5-Diaminopentane	0.76	0.13	1.43	1.68	1.35	1.55
	2.11	439	147	438	[M+H]+	N-(4'-O-glycosyl)-p-coumaroyl agmatine	0.69	0.66	1.13	1.24	0.77	0.58
	2.17	469	177	468	[M+H]+	N-(4'-O-glycosyl)-feruloyl agmatine	0.86	-0.44	1.46	2.48	1.38	2.92
	2.29	235	147	234	[M+H]+	N-p-Coumaroyl putrescine	1.42	3.77	0.414	0.281	1.26	-3.49
	2.29	265	177	264	[M+H]+	N-Feruloyl putrescine	1.48	2.61	1.29	1.56	1.27	-1.06
	2.38	235	147	234	[M+H]+	N'-p-Coumaroyl putrescine	1.41	3.72	0.329	0.253	1.25	-3.47
	2.77	295	207	294	[M+H]+	N-Sinapoyl putrescine	1.45	1.97	1.45	2.88	1.13	0.9
	2.87	321	177	320	[M+H]+	N-Feruloyl spermidine	1.42	2.04	1.21	1.34	1	-0.7
	2.95	309	207	308	[M+H]+	N-sinapoyl cadaverine	0.56	-1.72	0.842	2.22	1.11	3.94
	3.82	498	177	497	[M+H]+	N', N''-Diferuloylspermidine	1.5	1.15	1.46	2.22	1.35	1.07
	4.91	314	177	313	[M+H]+	N-Feruloyl tyramine	0.53	-0.11	1.08	2.88	1.04	2.99

Phenylpropanoids	2.39	351	105	352	[M-H]-	6,7-dihydroxycoumarin 7-O-quinic acid	1.05	0.85	1.03	-1.02	1.15	-1.86
	2.58	371	209	372	[M-H]-	Syringin	1.43	0.65	1.41	1.31	1.29	0.66
	2.72	339	177	340	[M-H]-	Esculin (6,7-Dihydroxycoumarin-6-glucoside)	1.34	0.72	1.46	1.45	1.24	0.73
	2.83	325	163	324	[M+H]+	Skimmin	1.52	1.59	1.47	3.67	1.35	2.09
	2.86	341	135	342	[M-H]-	Caffeic acid O-glucoside	1.29	0.7	1.33	1.05	1.03	0.35
	3.23	179	135	180	[M-H]-	Caffeate	1.37	-1.58	1.04	-0.97	0.92	0.61
	3.31	207	85	206	[M+H]+	6-Hydroxymethylherniarin	1.28	0.42	1.43	-1.69	1.38	-2.11
	3.31	785	623	786	[M-H]-	Echinacoside	1.2	-1.05	0.848	-0.39	0.95	0.66
	3.35	169	65	168	[M+H]+	Vanillic acid	1.52	-0.82	1.43	-1.07	0.86	-0.25
	3.39	741	579	742	[M-H]-	Eleutheroside E	0.49	-0.96	0.721	0.96	1.02	1.92
	3.67	149	131	150	[M-H]-	p-Coumaryl alcohol	1.4	1.54	0.865	-0.694	1.37	-2.24
	3.82	369	207	368	[M+H]+	N-sinapoyl hydroxycoumarin	0.47	0.11	1.41	-2.29	1.36	-2.4
	3.88	339	177	338	[M+H]+	O-Feruloyl 4-hydroxylcoumarin	1.02	0.78	0.93	-0.375	1.12	-1.15
	3.88	209	179	210	[M-H]-	Sinapyl alcohol	0.6	-2.49	0.681	0.735	1.07	3.23
	4.01	623	161	624	[M-H]-	Verbascoside	0.08	1.14	1.28	6.47	1.17	5.32
	4.06	225	147	224	[M+H]+	Sinapic acid	0.55	0.32	1.17	-0.795	1.15	-1.12
	4.95	591	367	592	[M-H]-	Disinapoyl hexoside	1.47	1.23	1.25	-1.52	1.32	-2.76
	5.63	147	119	148	[M-H]-	3,4-Dihydrocoumarin	1.15	2.09	1.01	1.06	1.22	-1.02
	8.45	355	103	354	[M+H]+	Notopterol	0.53	0.34	1.05	-1.92	1.01	-2.26
Terpenes and steroids	3.26	379	199	356	[M+H]+	Gentiopicroside	1.16	2.19	1.12	3.13	1.26	0.94
	3.33	359	197	358	[M+H]+	Sweroside	0.69	0.33	1.21	-1.36	1.33	-1.69
	5.14	425	305	424	[M+H]+	Ginkgolide B	1.57	12.09	1.48	12.8	0.93	0.71
	7.34	317	137	316	[M+H]+	Phytocassane D	1.16	2.26	0.501	1.11	0.72	-1.15
	7.62	779	617	780	[M-H]-	Saikosaponin d	1.34	-1.24	0.604	-0.377	0.96	0.86
	11.10	455	407	456	[M-H]-	Oleanolic acid	0.93	-0.71	1.33	-1.83	1.26	-1.12
Vitamins and derivatives	0.85	175	87	176	[M-H]-	L-ascorbate	1.44	1.53	0.193	-0.161	1.39	-1.69
	1.16	332	314	331	[M+H]+	Pyridoxine O-glucoside	1.44	-1.13	0.228	-0.0849	1.35	1.04
	1.16	170	134	169	[M+H]+	Pyridoxine	1	-0.5	1.29	-1.08	1.24	-0.58
	3.26	377	243	376	[M+H]+	Riboflavin	0.03	0.01	1.4	1.15	1.33	1.15
	3.73	138	78	137	[M+H]+	Nicotinic Acid Methyl Ester (Methyl Nicotinate)	1.5	0.66	1.37	1.33	1.08	0.67

[a] VIP, the variable importance in projection score;

[b] LogFC, log2(fold change);

[c] The isomers were shown in blue;

[d] -, the compound was not detected in the related samples.
